# The suitability of laboratory-bred *Anopheles cracens* for the production of *Plasmodium vivax* sporozoites

**DOI:** 10.1186/s12936-015-0830-0

**Published:** 2015-08-12

**Authors:** Chiara Andolina, Jordi Landier, Verena Carrara, Cindy S Chu, Jean-François Franetich, Alison Roth, Laurent Rénia, Clémentine Roucher, Nick J White, Georges Snounou, François Nosten

**Affiliations:** Mahidol Oxford Tropical Medicine Research Unit, Faculty of Tropical Medicine, Mahidol University, Bangkok, Thailand; Shoklo Malaria Research Unit, Faculty of Tropical Medicine, Mahidol University, Mae Sot, Thailand; Centre for Tropical Medicine and Global Health, Nuffield Department of Medicine, University of Oxford, Oxford, UK; Sorbonne Universités, UPMC Univ Paris 06, CR7, Centre d’Immunologie et des Maladies Infectieuses (CIMI-Paris), 91 Bd de l’Hôpital, 75013 Paris, France; Institut National de la Santé et de la Recherche Médicale, U1135, CIMI-PARIS, 91 Bd de l’Hôpital, 75013 Paris, France; Centre National de la Recherche Scientifique, ERL 8255, CIMI-PARIS, 91 Bd de l’Hôpital, 75013 Paris, France; Department of Global Health, College of Public Health, University of South Florida, Tampa, FL USA; Singapore Immunology Network (SIgN), Agency for Science, Technology, and Research (A*STAR), Singapore, Singapore

**Keywords:** *Anopheles cracens*, Sporozoites, *Plasmodium vivax*, Transmission, Insectary

## Abstract

**Background:**

A stenogamous colony of *Anopheles cracens* (*A. dirus B*) established 20 years ago in a Thai insectary proved susceptible to *Plasmodium vivax*. However, routine sporozoite production by feeding on field-collected blood samples has not been described. The setting-up of an *A. cracens* colony in an insectary on the Thai-Myanmar border and the process of using *P. vivax* field samples for the production of infectious sporozoites are described.

**Methods:**

The colony was started in 2012 from egg batches that were sent from the Department of Parasitology, Faculty of Medicine, University of Chiang Mai, to the Shoklo Malaria Research Unit (SMRU), on wet filter paper in sealed Petri dishes. From May 2013 to December 2014, *P. vivax*-infected blood samples collected from patients seeking care at SMRU clinics were used for membrane feeding assays and sporozoite production.

**Results:**

Mosquitoes were fed on blood samples from 55 patients, and for 38 (69 %) this led to the production sporozoites. The average number of sporozoites obtained per mosquito was 26,112 (range 328–79,310). Gametocytaemia was not correlated with mosquito infectiousness (p = 0.82), or with the number of the sporozoites produced (Spearman’s ρ = −0.016, p = 0.905). Infectiousness did not vary with the date of collection or the age of the patient. Mosquito survival was not correlated with sporozoite load (Spearman’s ρ = 0.179, p = 0.282).

**Conclusion:**

Consistent and routine *P. vivax* sporozoites production confirms that *A. cracens* is highly susceptible to *P. vivax* infection. Laboratory-bred colonies of this vector are suitable for experimental transmission protocols and thus constitute a valuable resource.

**Electronic supplementary material:**

The online version of this article (doi:10.1186/s12936-015-0830-0) contains supplementary material, which is available to authorized users.

## Background

*Plasmodium vivax* is the most widely distributed of the *Plasmodium* species infecting humans outside of Africa, with 80 million cases recorded yearly [1]. This species, characterized by a dormant liver stage, the hypnozoite, can resume its development and lead to relapses over the months or years following the initial infectious bite. Consequently, the effective duration of the infection and the number of carriers are significantly increased, which poses a serious obstacle to current efforts to control and eventually eliminate this parasite. Only one licensed drug, primaquine, is able to eliminate the liver stages of *P. vivax* [2]. Deployment of primaquine is severely restricted by its potential to cause haemolysis in persons with glucose-6-phosphate dehydrogenase deficiency that affects a significant proportion of the population in *P. vivax*-endemic countries [3]. This undesirable side effect characterizes other 8-aminoquinolines, including tafenoquine [4]. Thus, the development of an effective safer hypnozoitocidal drug has become urgent.

Screening for activity against hypnozoites has been restricted to relatively few compounds over the past 60 years, because it could only be conducted practically using macaques infected with *P. cynomolgi* [5]. This parasite that serves as a model for *P. vivax*, to which it is phylogenetically closely related, is one of the few that produces hypnozoites. The recent development of a protocol for the in vitro cultivation of *P. cynomolgi* hypnozoites opens the way to higher throughput screening [6, 7], as well as conducting investigations on the biology of the liver stages of relapsing parasite species. It is ultimately highly desirable to extend such investigations to *P. vivax* and to test for inhibitory activity against *P. vivax*. However, a regular supply of *P. vivax* infectious sporozoites is needed in order to develop and exploit hepatic stages in vitro cultivation protocols. This would rely on the simultaneous availability of susceptible anopheline mosquitoes and a supply of gametocyte-carrying infected blood. For *P. vivax*, a parasite that cannot at present be propagated in vitro, this implies the proximity of infected patient donors to a suitable anopheline colony.

The Shoklo Malaria Research Unit (SMRU), located on the Thai-Myanmar border, includes several clinics in which *P. vivax*-infected patients are treated, thus affording a regular supply of infected blood suitable for mosquito membrane-feeding. In Southeast Asia, some of the main malaria vectors belong to the *A. dirus* complex of the *Leucosphyrus* group [8] that comprises seven species of which five can be found in Thailand: *A. dirus* (*A. dirus* sensu stricto), *A. cracens* (*A. dirus* B), *A. scanloni* (*A. dirus* C), *A. baimaii* (*A. dirus* D), *A. nemophilous* (*A. dirus* F). These sibling species vary in behaviour and distribution as well as in their susceptibility to *Plasmodium* [9]. Given that most have a eurygamous mating behaviour (i.e. they mate only in large spaces), laboratory breeding requires repeated forced mating. About two decades ago a self-mating stenogamous colony of *A. cracens* (i.e. which can breed in confined spaces) was established in the insectary of the Department of Parasitology, Faculty of Medicine, at the University of Chiang Mai. Mosquitoes from this colony were found to be susceptible to infection by *P. falciparum* and *P. vivax* [10–12].

The establishment of a stenogamous colony of *A. cracens* at the SMRU insectary, in Mae Sot, Thailand, and routine sporozoite production from membrane-feeding experiments are described using *P. vivax*-infected blood samples collected over 20 months.

## Methods

### Study site

The experimental infections were carried out in the secure insectary at the SMRU, Mae Sot, on the western border of Thailand with Myanmar. Patients seeking care were admitted at SMRU migrant clinics located along the border, between 30 km north (Wang Pha) and 20–65 km south (Mun Ru Chai, Mae Kon Kaen and Mawker Thai) of Mae Sot, an area of low malaria endemicity [13].

### Ethics approval

The study was approved by Oxford Tropical Research Ethics Committee (Reference 28-09).

### Mosquitoes rearing

Rearing and maintenance of the mosquito colony was performed in a secure insectary maintained at 26 °C and a relative humidity of 80 %, with alternating 12 h cycles of light and dark. The secure insectary consists of four rooms. Infected mosquitoes are kept in locked incubators, which are separated from the outside by four doors. Access to the secure insectary is strictly restricted to authorized and trained personnel. The facility is fully sealed. The SMRU laboratories, including the insectary are part of the Mahidol University, Faculty of Tropical Medicine. Every 3 days mosquitoes were allowed to lay eggs on a Whatman filter paper wrapped in a conical shape and placed in a plastic bowl filled with 60 mL of distilled water.

The eggs were collected and rinsed with 1 % NaOCl, and then with distilled water on a new filter paper, using a modified Nalgene 500 mL disposable filter attached to a vacuum so as to minimize the contamination by microsporidia [14]. The eggs were then placed under a blue light bulb until hatching, which occurred within 2 days. One day later a sprinkle of fine TetraBits Complete^®^ food was added to the bowl. First instar larvae were transferred to plastic trays (25 cm × 36 cm × 6 cm) filled with 1.3 L of drinking water. The optimal number was around 60–70 larvae per tray. The water was changed every 2 days in order to minimize the risk of fungal or bacterial contamination. Trays with many dead larvae were discarded in order to select healthy mosquitoes. The larvae were fed twice daily with ground TetraBits Complete^®^, which had been cooked in an oven overnight at 121 °C and thereafter kept sealed in plastic bags in a refrigerator at 4 °C. On development, pupae were collected from the trays with a sterile syringe and transferred to small bowls filled with distilled water that were then placed inside cages until the emergence of adult mosquitoes. These were fed with cotton pads soaked with 5 % vitamins (MULTILIM syrup, Atlantic Laboratories Corp. Ltd, Bangkok, Thailand) and 10 % sugar solution. Every 4 days female mosquitoes were fed with human blood; either directly from an arm or by using a Hemotek^®^ membrane-feeding system using drawn heparinized blood. The number of adults that were maintained per 30 cm × 30 cm × 30 cm cage did not exceed 500. In order to maintain optimal humidity, wet clean towels were placed on the top of the cages that were then covered with a black plastic sheet.

### Mosquito colony

The *A. cracens* colony was considered stable 2 years after it was first initiated in the SMRU insectary. On average, 1,500–2,500 pupae were collected weekly and 6,000–10,000 adult mosquitoes were produced each month. Two cages of uninfected females were always kept to maintain the colony, and were fed every 3 days with uninfected blood. For each feeding experiment, it was estimated that only one-third of the mosquitoes actually ingested *P. vivax*-infected blood, those that did not feed were principally males. Mosquitoes were not recycled in the colony.

### Blood samples

Non-pregnant adult patients presenting with clinical symptoms (i.e., fever, chills, headache) and with a blood smear positive for *P. vivax* gametocytes were considered eligible for recruitment. After obtaining written consent, and prior to receiving anti-malarial drug treatment, 5–10 mL of heparinised venous blood were obtained and placed immediately in a water bath at 37–38 °C in order to avoid microgametocyte exflagellation and the emergence of macrogametes. Within 1 h post-collection, the blood samples were transported in thermos flasks filled with water at 37–38 °C from the clinics to the SMRU insectary. On reception, the samples were centrifuged at 1,800*g* for 5 min at 37 °C. Plasma was replaced with warmed AB^+^ serum, and the mixture transferred within 10 min to the insectary. The gametocytaemia was determined by counting the number of gametocytes per 500 white blood cells (WBC) by microscopic examination of Giemsa-stained thick blood smears, assuming an average of 8,000 WBC per µl as per WHO guideline [15].

### Mosquito infection

Four to seven day old female *A. cracens* mosquitoes starved for 6–7 h were selected for feeding and placed in a small cup covered with netting material (up to 30 mosquitoes per cup). The mosquitoes were allowed to feed in the shade for 30 min to 1 h using a Hemotek^®^ feeder at 38 °C. Those that fully fed were moved from the small cup to a plastic container (up to 60 mosquitoes per container). Cotton pads soaked with a 10 % sugar solution were placed on top of the containers and they were changed daily. The containers were kept at 26 °C with a relative humidity of 80 %. Vitamins were not provided.

### Oocysts count

Three to 22 mosquitoes from each feeding experiment were selected 7–8 days post-infection for midgut dissection in order to assess the presence of oocysts. If all three proved negative for oocyst, the batch was considered poorly infected and this was at times confirmed by dissecting more mosquitoes. The midguts were stained with 1 % Mercurochrome and examined under a light microscope at 40× (total magnification) to enumerate oocysts.

### Sporozoites count

Between days 15 and 21 post-infection all the remaining fed mosquitoes were collected in small cups, anesthetized by placing at 4 °C and then killed in 70 % ethanol. After rinsing twice with sterile medium (Leibovitz or RPMI), the salivary glands were dissected under a stereomicroscope, and then pooled together in a 1.5-mL Eppendorf tube with medium, in which they were crushed with a sterile pestle. After centrifugation, 10 µL of a sporozoites suspension was placed into a KOVA^®^ Glassic ^®^ Slide 10 with grids or into a Neubauer chamber haemocytometer. A phase-contrast microscope set to 40× magnification was used to enumerate the sporozoites. The average number of sporozoites counted per grid was multiplied per the dilution factor and multiplication factor of the chamber in order to calculate the sporozoites per µL.

### Mosquito survival rate after infection

The survival rate was calculated by dividing the number of mosquitoes still alive 2 weeks after infection by the original number of fed mosquitoes excluding the mosquitoes used for midgut dissection.

### Data analysis

All analyses were performed using Stata 12.0 (StataCorp, College Station, TX, USA). Gametocytaemia values were log-transformed to obtain a normal distribution and Student’s *t* test was used to compare mean log-gametocytaemia between infectious and non-infectious gametocytaemic blood. Results are presented as geometric means of original values for each group. Sporozoite production was evaluated by the arithmetic mean number of sporozoites obtained per mosquito dissected from a given batch. Spearman’s correlation coefficient was used to assess the relationships between gametocytaemia and sporozoite production, and between gametocytaemia and mosquito mortality in infected mosquito batches; Mann–Whitney test was used to analyse if transmitters and non-transmitters differed in their age distribution.

### Establishment of the *Anopheles cracens* colony

The initial attempts to establish a colony at the SMRU were made at the beginning of 2012 from egg batches sent from the insectary of the Department of Parasitology, Faculty of Medicine, University of Chiang Mai (Thailand), to the SMRU on wet filter papers in sealed Petri dishes. A number of factors (type and temperature of the water, number of larvae per tray) needed to be optimized in order to achieve optimal adult production.

As it was not possible to use the same source of water that was used in Chiang Mai, different types of water were tested at the SMRU: natural fresh spring water collected directly from the source, filtered water left for a few days in a tank to let the chlorine evaporate, de-ionized water mixed with spring water, or mineral drinking water. Satisfactory full larval development was obtained only when mineral drinking water was used. The larvae were maintained in tray with 1.3 L of water, and optimal development and minimal mortality were obtained at densities not exceeding 80 larvae per tray. The larvae were fed with fine powdered fish food, with a small amount (0.01 g) sprinkled twice per day. In order to minimize the risk of contamination by fungi and bacteria, aquatic macrophytes were not used, and the larvae were moved to new trays filled with clean water every 2–3days as surface scum became obvious. The optimal water temperature was found to be 25 ± 1 °C, and small lamps with 40-W lights were shone on the trays to maintain this temperature. Under these conditions the cycle of development to pupae lasted about 15 days.

*Anopheles cracens* have been shown to preferentially feed on human blood. Attempts to feed adults on the blood of white rats, mice, guinea pigs, gerbils or rabbits proved unsatisfactory. Indeed the original colony had been maintained on human blood from volunteers for many decades in the laboratory and it was considered safe to feed the mosquitoes directly on human arms during the initial phases of colony establishment at the SMRU. As the colony grew, direct feeding was replaced with artificial membrane feeding using human heparinized blood. This human blood was kept in the refrigerator, and renewed every week.

The main danger that faced the colony, at SMRU and at Chiang Mai, was microsporidial infection. This was detected by observing spores in the midgut of dissected mosquitoes under a light microscope at 100×. The infection led to high pupal mortality, reduction in egg production and a consequent dramatic decrease in colony size. More importantly, afflicted mosquitoes lost their susceptibility to infection by *Plasmodium* and those that fed on malaria-infected blood showed increased mortality, in a similar manner to that reported in the literature [16, 17]. Thus, strict measures were necessary to control and eliminate any microsporidial contamination. The whole insectary was cleaned with soap and water, and glass shelving was used, as it was easier to clean. The cotton pads soaked with sugar and vitamins solution, and the water to prepare it were autoclaved before use. Aliquots of TetraBits^®^ fish food were cooked overnight at 121 °C in order to kill the spores, then sealed in clean plastic bags, and kept at 4 °C for not more than 3 days. The food stock was kept at −20 °C until use. Gloves were worn to handle larval and adult food. Given that microsporidial spores can spread from generation to generation through horizontal and vertical transmission (depending on the species) or through cannibalism of infected larvae [18], the trays were monitored for dead larvae. Dead larvae were removed, dissected and examined for microsporidial spores, and the trays where any were found removed and sterilized.

Mosquito eggs were bleached with 1 % NaOCl for 1 min and then rinsed with sterile water, the larval trays were rinsed with boiled water and 70 % ethanol, and the cages’ netting material was replaced. The pupae were rinsed twice in deionized water before the emergence of adults and the towels covering the mosquito cages were autoclaved and subsequently rinsed daily in boiled water. Full recovery of a colony after microsporidial infection took 12 months to achieve.

## Results

### Assessment of infectivity to *Plasmodium vivax* field strains

From May 2013 to December 2014, a total of 55 *P. vivax*-infected patients with microscopically detectable circulating gametocytes were identified. Informed written consent was sought and obtained for the provision of blood samples for mosquito feeding. About 8,000 female mosquitoes successfully ingested blood meals from these samples via the membrane feeding system.

The number of mosquitoes available on reception of a blood sample was variable, thus the batches of mosquitoes that actually fed on a given blood sample varied from 23 to 459. The ultimate goal of the feeding experiments was to provide the large numbers of sporozoites required for in vitro infections of hepatocytes. Thus, in order to maximize the number of mosquitoes available for salivary gland dissection about 14 days post-feeding, it was decided to ascertain the infective status of the batch fed on each sample by dissecting a small number of mosquitoes (in most cases three mosquitoes) on day 7 to assess the presence of oocysts. If none were positive, the batch was considered likely to have been poorly infected and considered negative. In this manner, 17/55 blood samples (31 %) were considered to have been poorly infectious or non-infectious to mosquitoes and consequently discarded. These patients are denoted as non-transmitters. For the remaining 38 (68 %) blood samples oocysts were observed in at least one of the fed mosquitoes that were dissected (in all mosquitoes for 32 of the samples), and these patients are referred to as transmitters.

**Fig. 1:**
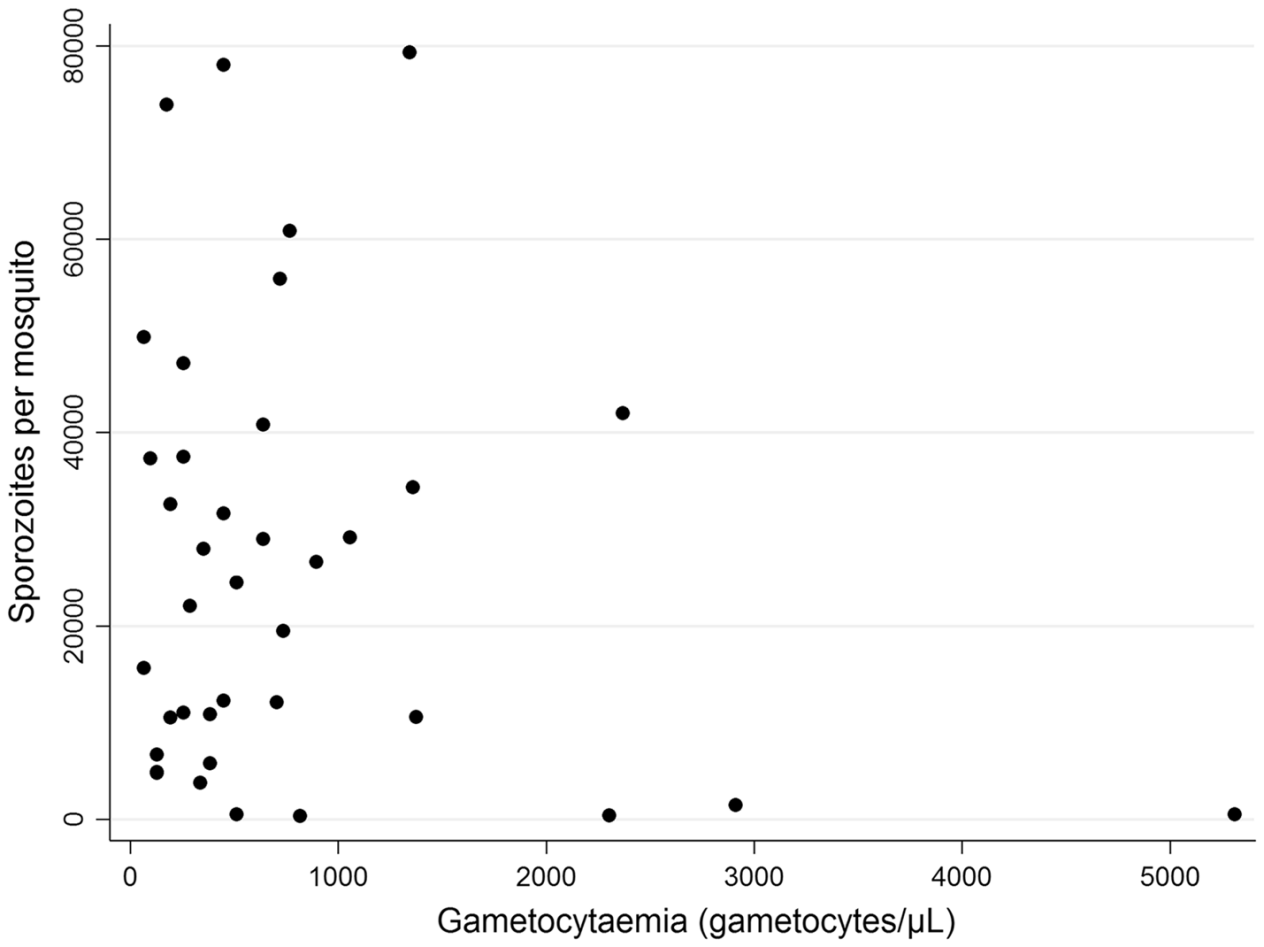
Relationship between *Plasmodium vivax* gametocytaemia (x-axis) and mean number of sporozoites per mosquito (y-axis).

The gametocytaemia recorded did not correlate with infectivity to mosquitoes [geometric mean gametocytaemia: 428 (201–908) for the non-transmitters (n = 17) versus 464 (330–652) for the transmitters (n = 38); p = 0.817]. The numbers of oocysts observed per mosquito varied from 1 to 200, however, the low number of mosquitoes dissected for each batch was not sufficient for any meaningful analysis of the relationship between the number of oocysts and the gametocytaemia or the number of sporozoites produced. For the 38 transmitters, gametocytaemia did not correlate with sporozoite production (Spearman correlation coefficient between gametocytaemia and mean number of sporozoites per mosquito (ρ = −0.0793; p = 0.6361) (Fig. 1).

In the 38 infected mosquito batches, the sporozoite production per batch (defined as the mean number of sporozoites per mosquito dissected from a given batch) varied from 328 to 79,310 sporozoites per mosquito, exceeding 10,000 in 28 batches. Across all infective batches, the mean production was 26,112 sporozoites per mosquito dissected (range 328–79,310).

**Fig. 2:**
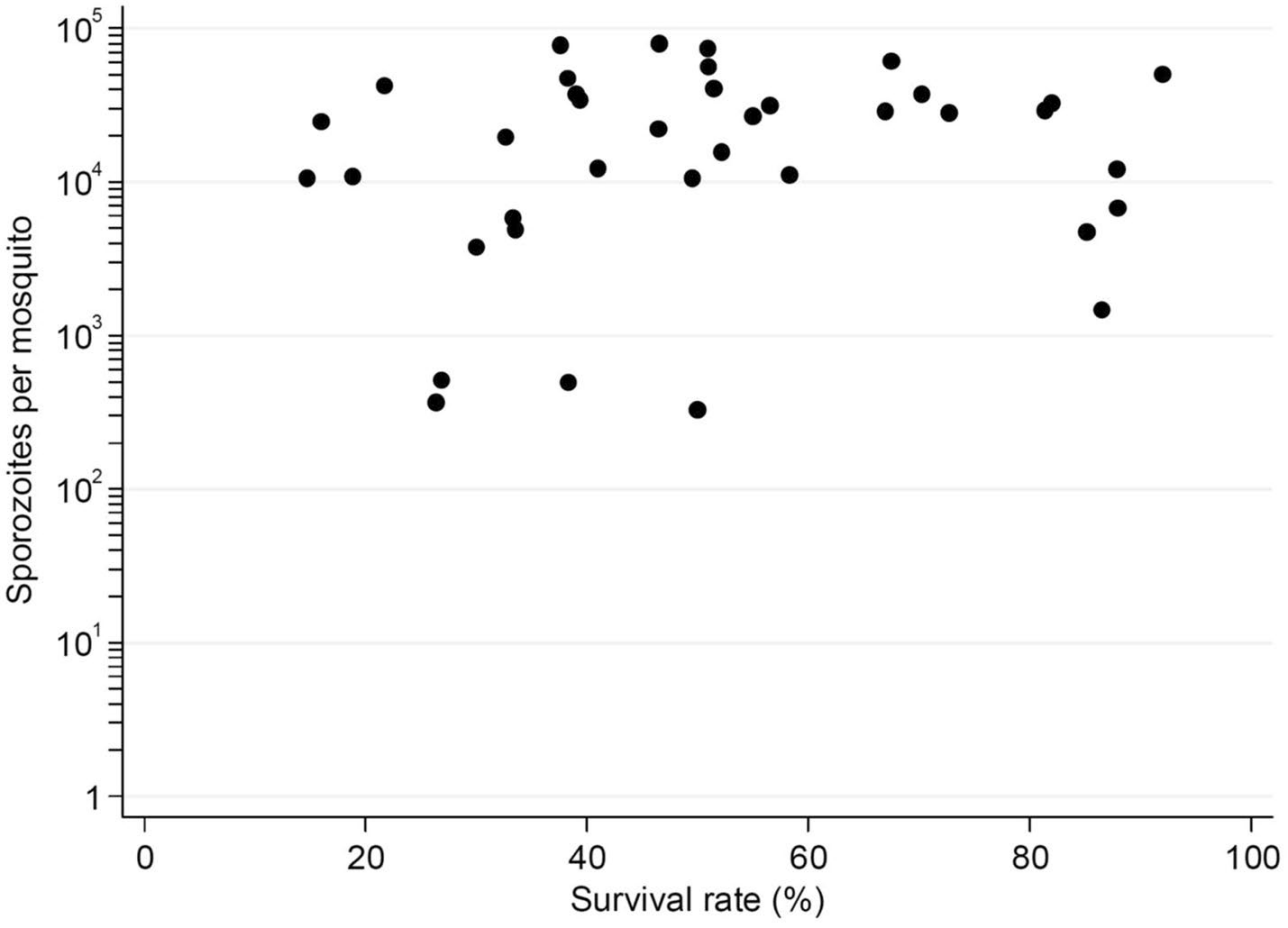
Relationship between mosquito survival (% x-axis) and mean number of sporozoites per mosquito (y-axis).

Samples were mainly collected from young adults (median age [Interquartile range] = 20 [15–34], n = 54) and no significant difference was found in age distribution among transmitters and non-transmitters (p = 0.545). Mosquito mortality at day 14 post feeding was variable for the batches that became infected (Fig. 2), but it did not correlate with the mean number of sporozoites per mosquito (Spearman’s ρ = 0.179, p = 0.282). The overall mortality for all the mosquito batches that became infected was 46 %. The dataset from on which these analyses were based is provided in Additional file 1.

## Discussion

In the early 1980s, a colony of *A. cracens* (previously known as *A. balabacensis*, then as *A. dirus* B) was made to adopt stenogamous behaviour under laboratory conditions, making it suitable for breeding in captivity [19]. This colony was shown to be susceptible to transmit *P. vivax* [10–12], although these observations were limited to very few *P. vivax* samples (three, one, and three, respectively) and the sporozoites produced were not quantified. In this study, the blood from infected *P. vivax* patients living along the Thai-Myanmar border was used to ascertain this species’ vectorial capacity. To this end, an *A. cracens* colony was established and used for the experimental transmission of 55 distinct *P. vivax* samples collected over a 20-month period (May 2013 to December 2014) spanning successive two-yearly transmission peaks (June to August and October to November). Over the study period a total of about 91 million *P. vivax* sporozoites were obtained at the SMRU, with a total of more than a million sporozoites obtained from each of 21 patient samples out of the 38 that were considered as transmitters. It should be noted that for 29 of the patients, the number of mosquitoes available for salivary gland dissection was less than 100, yet for 14 of these more than a million sporozoites were obtained.

Thus, the data obtained indicate that female *A. cracens* are highly susceptible to infection by *P. vivax*, with about 70 % of the subject samples leading to the production of salivary gland sporozoites. For the purposes of this study it was not deemed necessary or desirable to use molecular methods to detect or quantify gametocytes. It was important that the blood sample was conveyed to the laboratory for the feeding with the minimum of delay. Thus, sample selection was based on the microscopic observation of gametocytes at the time of admission. Gametocytaemia was not significantly different between transmitter and non-transmitter batches, and it was also not significantly correlated with the mean number of sporozoites per mosquito obtained for a given batch. This pattern was observed for similar studies conducted in Thailand [20] and Colombia [21], but not for others conducted in Brazil [22] and Peru [23, 24]. However, it should be noted that differences in a number of important factors make it difficult to draw any conclusions from such comparisons. In addition the parasites originated from different geographical locations and the species of mosquitoes used differed between the studies; both have been known to affect infection outcome [25]. Moreover, it has previously been noted that infectivity to mosquitoes varies with the course of infection, and possibly between primary and relapse episodes [26–31]. Thus, heterogeneity in admission samples could account for most of the variations observed.

One measure relevant to the utility of experimental transmission is the mean number of sporozoites per fed mosquito. Indeed, laboratory investigations on sporozoites and in particular on the liver stages require large numbers of parasites (e.g., 25,000 sporozoites are generally used to infect the hepatocytes in a single well of a 96-well plate, and data for each experimental point are generally derived from triplicate wells). For 28 of the 38 transmitter samples, the mean number of sporozoites per surviving fed mosquito exceeded 10,000. The overall mean number of sporozoites per mosquito obtained for the *A. cracens* at SMRU (26,112; range 328–79,310) compares favourably with that observed for *A. dirus* A (Bangkok colony) maintained in Thailand (9,525; range 0–285,000). However, this mosquito line, that has provided significant insights into the transmission biology of *P. vivax* [20, 32, 33], requires labour-intensive, forced-mating for its maintenance.

## Conclusion

The free-mating *A. cracens* colony established more than 30 years ago by Sucharit and Choochote [16], was amenable to expansion in another site where it was shown to be suitable for efficient transmission of field-collected *P. vivax* and routine production of relatively large numbers of sporozoites, thus providing a valuable resource for future research on this hitherto neglected important human pathogen.

## Additional file

Additional file 1: Raw data on mosquito’s infections and sporozoites production. This file shows the original data analysed and displayed in the graphs in the present manuscript.
